# COVID-19 pandemic’s burden on healthcare professionals’ mental health

**DOI:** 10.1192/j.eurpsy.2021.720

**Published:** 2021-08-13

**Authors:** O. Khaustova, O. Chaban, D. Assonov

**Affiliations:** Medical Psychology, Psychosomatic Medicine & Psychotherapy, Bogomolets National Medical University, Kiyv, Ukraine

**Keywords:** COVID-19, mental health, Healthcare professionals, resilience

## Abstract

**Introduction:**

Healthcare professionals report about anxiety, depression, and fear during pandemic COVID-19 worldwide. Resilience becomes the high-powered important mechanism that reduces stress impact on the emotional state of healthcare professionals.

**Objectives:**

We suggested that effective resilience is associated with less COVID-19’s fear, as well as less anxiety, and depression; healthcare professionals’ mental health depends on age, gender, as well as involvement in the care of patients with COVID-19.

**Methods:**

211 healthcare professionals participated in the study and were evaluated with the Connor-Davidson Resilience 10-item scale (CD-RISC-10), Fear of COVID-19 Scale, PHQ-9, GAD-7.

**Results:**

A negative correlation between resilience and fear of COVID-19 (p≤0,01), anxiety (p≤0,01), and depression (p≤0,001) was found. Positive correlations were found between depression, anxiety, and fear of COVID-19 (p≤0,001), between age and fear of COVID-19 (p≤0,05). No statistically significant association between age and depression, anxiety, or resilience was found. The significant difference of COVID-19 fear depending on gender – female vs male (p≤0,05) was found. No statistically significant difference in resilience and emotional state in healthcare professionals depending on the involvement in the care of patients with COVID-19 were found.

**Conclusions:**

Resilience is associated with better mental health in healthcare professionals during the COVID-19 pandemic. Anxiety and depression are connected with the fear of COVID-19 and highly comorbid in healthcare professionals. The elder age and female gender are among the risk factors for a more deteriorated mental state. Fear of COVID-19, mental state, and resilience are not associated with healthcare professionals’ involvement in the care of patients with COVID-19.

